# Yellow Fever at Norfolk

**Published:** 1855-11

**Authors:** 


*Yellow Fever at Norfolk, Va.*—We clip the following ni relation to those members of the profession who went to the aid of the suffering people of Norfolk, during the recent fatal epidemic from the *Daily Tribune*, of this city. It is no more than a just acknowledgement of the patriotism and philanthrophy of our profession:

HEROISM AT NORFOLK, VA.

Many a man who would plant a flag on a bastion, regardless of the winged messengers of death which fly thick and fast around him, dare not face it when it comes in the form of a pestilence. Yet they who brave disease, when the very air is full of poison, and contagion sweeps it from house to house, are the heroes of the day. None may compare with them! Not they, certainly, whose hands are red with blood of the battle-field, or who wear a crown wet with it.

The Southern *Argus*, a Norfolk journal, after a suspension of thirty-nine days, re-appears, and gives, in its first issue, a picture of the "doom and devastation of the plague." It raged for near three months. During this period, in an average population of six thousand, with few exceptions, every man, woman and child, was "striken with the fall fever," and about two thousand have been buried—"being not less than two out of three of the whites, and one out of three of the whole abiding community of Norfolk, white and black." Where else, or when before, has the pestilence smitten any community with a harsher doom? It is terrible to think on it.

Mr. Whitehead, acting Mayor, writes to the physicians who met the pestilence and did their duty, as follows:

"Had we not received material aid from abroad—had not the different portions of our country sent their heroic delegations of physicians, nurses and stalwart co-laborers—had not noble spirits volunteered to the rescue, (to die if need be, like Curtius, for Rome), our people must have sunk beneath the burthen of their agony. There was a period (about the first of September) when the evil seemed greater than we could bear. Corpses lay unburied—the sick unvisited—the dying unanealed. Our surviving physicians were either sickening or becoming exhausted, and our remaining population was panic-struck at the sight of accumulating horrors and duties. You who came to our relief were astounded at the unrealized state of things which you found here—an evil, the like of which you had never before witnessed. But nerving yourselves to the task, and telegraphing for reserves, you went resolutely forward with your science and its accompaniment, carrying aid where it was most needed, and infusing vigor into may hearts that would otherwise have soon ceased their painful throbbing."

The names of these physicians, the dead and the living, should be as household words—for they are heroes of whom the world should be proud. No nobler list of good men can any country's record present.

We give first the names of those who escaped the pestilence:

Dr. W. Stone, New Orleans, Aug. 16, 1855; Dr. Thos. Penniston, New Orleans, Aug. 17; Dr. Wm. H. Freeman, Philadelphia, Aug. —;

Rev. T. G. Keen, Petersburgh, Aug. 20; Dr. De Castro, Cuba, Aug 21; Dr. John F. Carter, Richmond, Aug. 23; Dr. John Morris, Baltimore, Aug. 24; Capt. Nathan Thompson, Philadelphia, Aug. 24; Dr, A A Zeiglfuss, Philadelphia, Aug. 25; Dr. Jas. Mc'Fadden, Philadelphia, Aug. 26; Dr. Randall, Philadelphia, Aug. 25; Dr. J. T. Hargrove, Richmond, Aug. 25; Dr. E. D. Fenner, New Orleans, Aug. 25; Dr. C Beard, New Orleans, Aug. 25; Dr. E J. Worl, Philadelphia, Aug. 25, Dr. St J. Ravsnel, S. C. Aug. 27; J. N. Crow, Richmond, Aug. 28; Dr. A. B. Williams, S. C. Aug. 28; Dr. Covert, S. C. Aug. 28; Dr. Rich, S C. Aug. 28; Dr. J. Hitt, Georgia, Aug. 20; Dr. W. H. Hugar, S. C. Aug. 29; Dr. T. C. Skrine; S. C. Aug. 29; Dr. F. M. Garret, N. C. Aug. 26; A. M. Loryear, S. C. Aug. 29; A. R. Taber, S. C. Aug. 29; Dr. Bignon, Georgia. Aug. 29; Dr. Donaldson, Georgia, Aug. 29; A. J. Gibbs, Philadelphia, Aug. 30; Dr. Marsh, Philadelphia, Aug. 30; Dr. E. C. Steele, S. C. Aug. 20; W. Porcher Miles, S. C. Aug. 30; Dr. Campbell, New Orleans, Aug. 30; D. I. Ricardo, New Orleans, Aug. 30; Dr. J. B. Read, Georgia, Aug. 30; Dr. Godfrey, Georgia, Aug. 30; Dr. Skinner, Georgia, Aug. 30; Dr. Charson, Georgia, Aug. 30; Dr. Mc Farland, Georgia, Aug. 30; Dr. Nunn, Georgia, Aug. 30; Capt. Thos. J. Ivy, New Orleans, Aug. 30; E. E. Jackson, S. C. Aug. 30; Dr. Williams, D. C. Aug. 31; Dr. G. S West, N. Y. Aug. 31 ; Dr. J. B. Holmes, S. C. Aug. 31; Judge Olin, Georgia, Sept. 1; John Tallaferro, Georgia, Sept. 1; Dr. Freer, N. Y. Sept. 1; Franklin H. Clark, New Orleans, Sept. 5; Dr. Robinson, N. Y. Sept. 3; Dr. R. M. Miller, Mobile, Sept. 3; Wm. Ballantyne, Mobile, Sept. 3; W. B. Thompson. Georgia, Sept. 6; Dr. Baker, Key West, Sept. 7 ; W. T Waithall, Mobile, Sept. 7; Dr. Flournoy, Arkansas, Sept. 8; Dr R. R. McKay, Georgia Sept. 9; Dr. A. B. Campbell, Philadelphia, Sept. 9; Dr, Wilson, Cuba, September 11 ; William C. Miller, Mobile, September 12; William N. Ghiselin, New Orleans, September 12 ; Mr. Rucker, Montgomery, Ala. Sept. 13; Mr. Clows Montgomery, Ala. Sept. 13; Dr. Fredricks, N. Y. Sept. 14. Dr. John Vaughan, London, Sept. 17; Dr. McFarlane, N. Orleans, Sept. 18; A. H. Jennet, Mobile, Sept. 18.

Those who fell—the blessed martyrs—are :

Dr. Leon Gilbert, dead, Richmond, Aug. 22; Dr. P. C. Gooch, dead, Richmond, Aug. 22; Dr. Walter, dead, Baltimore, Aug. 23; Dr. Robert Thompson, dead, Baltimore, Aug. 24; Dr. T. H. Craycroft, dead Philadelphia, Aug. 24; Dr. Fliess, dead, Baltimore, Aug. 24; Dr. T. Booth, dead Baltimore, Aug. 25; Dr. Howe, dead, Baltimore, Aug. 25; Dr. Howle, dead, Richmond, Aug. 25; Dr. T. P. McDowell, dead, Richmond, Aug. 25; Dr. T. Mierson, dead, Philadelphia, Aug. 26; Dr. Richard Blow, dead, Sussex, Vt. Aug. 28; Dr. Thomas, W. Handy, dead, Philadelphia, Aug. 26; Dr. C. A. Smith, Pa. Aug. 30; Dr. Jackson, dead, D. C. Aug. 31; Dr. Dabereshe, dead, D. C. Aug. 31; Dr. Schell, dead, N. Y. Aug. 31; Dr. Obermuller, dead, Ga. Sept. 5; Dr. R. B. Berry, dead, Tennessee, Sept. 8; Dr. Dillard, dead, Montgomery, Ala. Sept. 13; Dr. Capry, dead, N. Y. Sept. 14.

